# The present state of treatments for articular cartilage defects in the knee

**DOI:** 10.1308/003588412X13171221592573

**Published:** 2012-09

**Authors:** JR Perera, PD Gikas, G Bentley

**Affiliations:** Royal National Orthopaedic Hospital NHS Trust,UK

**Keywords:** Autologous chondrocyte implantation, Osteochondral lesions, Chondral lesions, Mosaicplasty, Matrix assisted chondrocyte implantation, Scaffolds, Tissue engineering

## Abstract

**INTRODUCTION:**

Chondral and osteochondral lesions of the knee are notoriously difficult to treat due to the poor healing capacity of articular cartilage and the hostile environment of moving joints, ultimately causing disabling pain and early osteoarthritis. There are many different reconstructive techniques used currently but few are proven to be of value. However, some have been shown to produce a better repair with hyaline-like cartilage rather than fibrocartilage.

**METHODS:**

A systematic search of all available online databases including PubMed, MEDLINE® and Embase™ was undertaken using several keywords. All the multiple treatment options and methods available were considered. These were summarised and the evidence for and against them was scrutinised.

**RESULTS:**

A total of 460 articles were identified after cross-referencing the database searches using the keywords. These revealed that autologous and matrix assisted chondrocyte implantation demonstrated both ‘good to excellent’ histological results and significant improvement in clinical outcomes.

**CONCLUSIONS:**

Autologous and matrix assisted chondrocyte implantation have been shown to treat symptomatic lesions successfully with significant histological and clinical improvement. There is, however, still a need for further randomised clinical trials, perfecting the type of scaffold and the use of adjuncts such as growth factors. A list of recommendations for treatment and the potential future trends of managing these lesions are given.

Articular (hyaline) cartilage (AC) has two main functions: low friction movement and shock absorption. Its varying properties enable it to comply with these functions. With increasing activity in young people and the longevity of older people, the prevalence of disorders affecting AC is increasing.^1^ Normally, the repair of chondral and osteochondral lesions is by fibrocartilage from blood released from the bone marrow. This contains undifferentiated mesenchymal stem cells (MSCs), which produce fibrocartilage containing predominantly type I and III collagen with abnormal proteoglycans. These generally give inadequate mechanical properties, leading to cartilage breakdown and, often, early osteoarthritis.

## Methods

All the available online databases including PubMed, MEDLINE® and Embase™ were searched for several keywords: autologous chondrocyte implantation, matrix assisted chondrocyte implantation, osteochondral and chondral defects, mosaicplasty and cartilage scaffolds as well as their common abbreviations. These were cross-referenced with ‘knee’. There was no restriction on publication date. This search yielded 460 articles on the management of knee cartilaginous defects, which were then reviewed.

## Structure of articular cartilage

**Figure 1:**
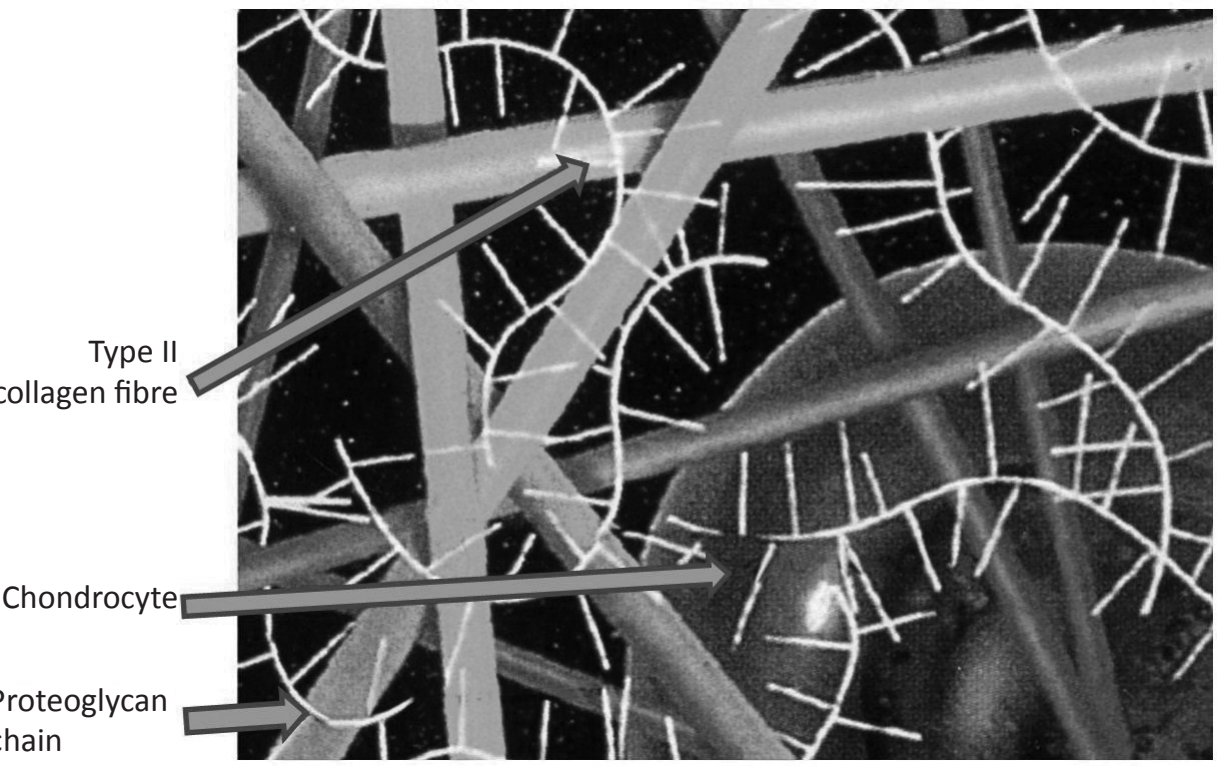
Spatial relations of collagen, proteoglycans and cells in cartilage

AC has a unique extracellular meshwork predominately of a type II collagen scaffold containing mainly water (approximately 70–80%) with the remainder being hydrophilic proteoglycans. The proteoglycans are made up of chondroitin sulphate and keratin sulphate and attach via a protein core to hyaluronan, forming a three-dimensional structure. These hold water within the collagen meshwork by their negative charge. The collagen meshwork is made up of 90% type II collagen with the remainder being type V, VI, IX and XI collagen.^2,3^ Type II collagen is strong in tension which, together with the contained water, gives the cartilage its toughness and resiliency (Fig 1).

The chondrocytes are spread sparsely throughout the AC and have differing functions. In the superficial tangential zone they are small and flattened and produce lubricin, for boundary lubrication. In the middle zone they are round and arranged into columns giving resiliency for shock absorption. Thus, synovial joints have a coefficient of friction of 0.0053, far less than the lowest friction prosthetic joint.^4^

## Metabolism and repair capability

AC is an avascular, aneural tissue with anaerobic metabolism and therefore has limited repair potential. In addition, chondrocytes have no significant migratory ability as they are embedded in the collagen matrix. Finally, the continuous use of the extremity with shearing and impact loading by the individual produces repetitive forces through any given lesion. This adds an unfavourable mechanical environment for spontaneous repair, eventually predisposing the individual to the development of osteoarthritis. The severity of damage is commonly graded using the Outerbridge classification (Table 1).^5^

**Table 1:** Outerbridge classification of articular cartilage defects^5^

Grade	Description
0	Normal
I	Cartilage with swelling and softening
II	Loss <50% cartilage thickness without exposure of subchondral bone
III	Loss <50% cartilage thickness without exposure of subchondral bone
IV	Complete loss of cartilage with subchondral bone exposure

In a retrospective review of more than 31,000 arthroscopic procedures, Curl *et al* found a 63% prevalence of chondral lesions with an average of 2.7 lesions per knee.^6^ A prospective study of 1,000 consecutive arthroscopies demonstrated some type of chondral or osteochondral lesion (OCL) in 61% of the patients and focal chondral or osteochondral defects were found in 19%.^7^ In other studies, the prevalence of focal articular lesions has been reported to be as high as 22–50%.^8^

## Osteoarthritis

Osteoarthritis is characterised by progressive AC loss, appositional new bone formation and sclerosis of the subchondral bone trabeculae, formation of marginal osteophytes and an imbalance between loss of cartilage resulting from matrix degradation and any attempt to repair this matrix. Despite major progress in the last few years, the aetiology, pathogenesis and progression of this disease are poorly understood. However, longstanding traumatic loss of AC is a well recognised predisposing risk factor for osteoarthritis.

Various methods have been used by orthopaedic surgeons to manage patients with severe and persistent pain caused by osteochondral injury. Many of these, like debridement, drilling, abrasion chondroplasty, microfracture and the insertion and use of carbon fibre pads, aim to induce only fibrocartilaginous reparative tissue, formed from primitive MSCs in the subchondral bone marrow. Other treatment strategies aim for repair with hyaline cartilage (AC cell autografting and osteochondral allografts).

In 1971 Bentley and Greer first showed that isolated chondrocytes could be used to repair articular surfaces of rabbit knees that had osteochondral defects or experimental arthritis.^9^ In 1982 Aston and Bentley showed that chondrocytes could be grown and multiplied by long-term culturing of cells at high density while maintaining the normal type II collagen and proteoglycans of the matrix.^10^ Cells were also grown and produced type II collagen and proteoglycans in a matrix of carbon fibre, leading to the potential for clinical application.^11^

Today, autologous chondrocyte implantation (ACI) is a treatment option for full-thickness chondral or osteochondral injuries that are painful and debilitating. Goals of surgery and rehabilitation include replacement of damaged cartilage with hyaline or hyaline-like cartilage, leading eventually to an improved level of function. Intermediate and long-term results are promising in terms of function and prevention of osteoarthritis.^12–15^

## Indications for surgery

Patients with a symptomatic OCL in a joint (that is otherwise normal) of the femoral condyles/trochlea or the patella are recommended for ACI.^16^ For larger defects (1–12cm^2^) ACI should be considered.^15^ Smaller lesions (<1cm^2^) can be managed initially using mosaicplasty or microfracture. If this fails, then ACI should be considered.^17,18^

A well motivated and compliant patient between the ages of 15 and 55 years who has had an OCL for under a year and no previous surgery is the best candidate. A careful and comprehensive general and lower limb orthopaedic physical examination with standard weight bearing anteroposterior and patellofemoral x-rays are mandatory.^19,20^ Reciprocal (kissing) lesions are a contraindication to ACI.^21^ Malalignment of the tibiofemoral and patellofemoral joints with or without a cruciate ligament injury can be corrected at the time of surgery. Osteoarthritis and inflammatory arthritis are also contraindications.

## Investigations

**Figure 2:**
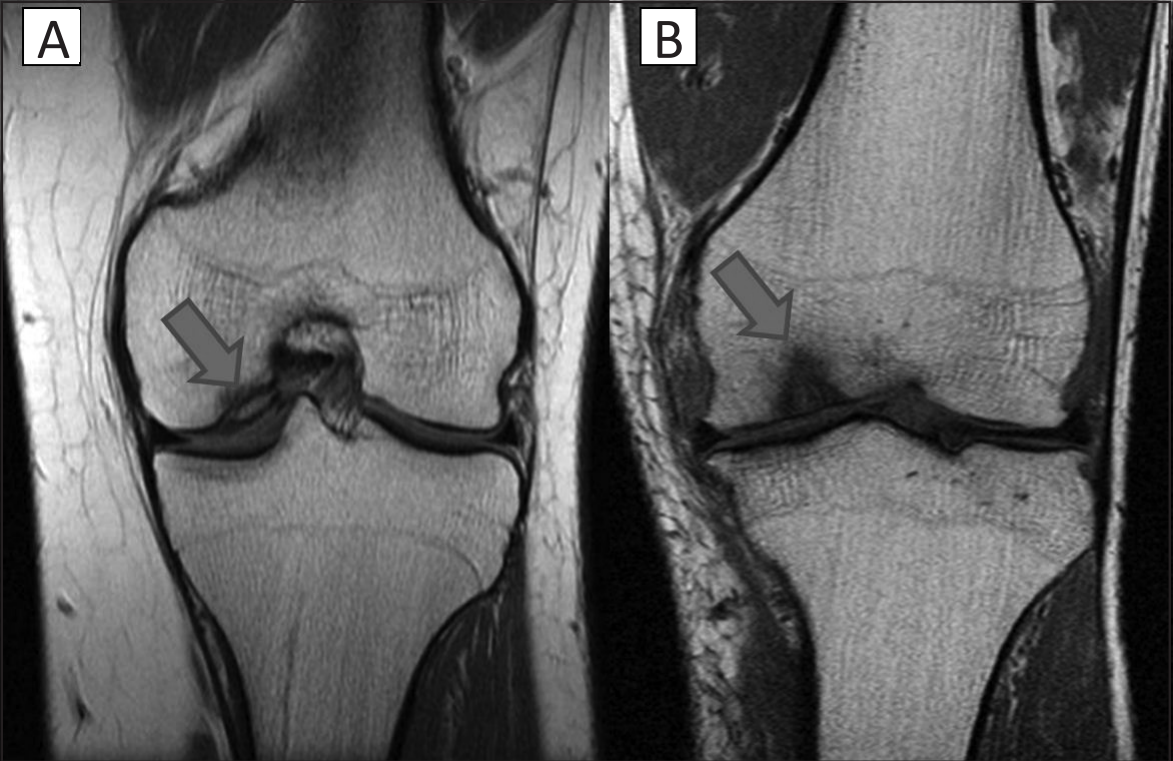
Coronal T1 weighted magnetic resonance imaging of the knee showing an area of osteochondritis dissecans affecting the medial femoral condyle (A) and an osteochondral lesion affecting the medial femoral condyle (B)

Until recently, the gold standard for investigation of the knee was arthroscopy. However, in 2008 von Endelhardt showed that high powered (>3T) magnetic resonance imaging (MRI) can be as reliable except in differentiating between grade II and III lesions (Fig 2).^22^ Furthermore, it can be used to assess the soft tissues around the knee as well as the surgical repair of defects.^23^ Cruciate ligament and meniscal pathology detected on MRI should be dealt with either before or at the time of dealing with the OCL.

## Choice of procedure

### Abrasion and drilling

This is arthroscopic debridement or low speed drilling, using a fine (1–2mm) K-wire, of the OCL to directly stimulate release of stem cells from the underlying bone marrow. MSC stimulation results in 22% of type I collagen (fibrotic tissue), 30% of degenerated hyaline cartilage and 28% of fibrocartilage.^24^ This method is now used for very small lesions.

### Microfracture and ‘marrow stimulating’ techniques

**Figure 3:**
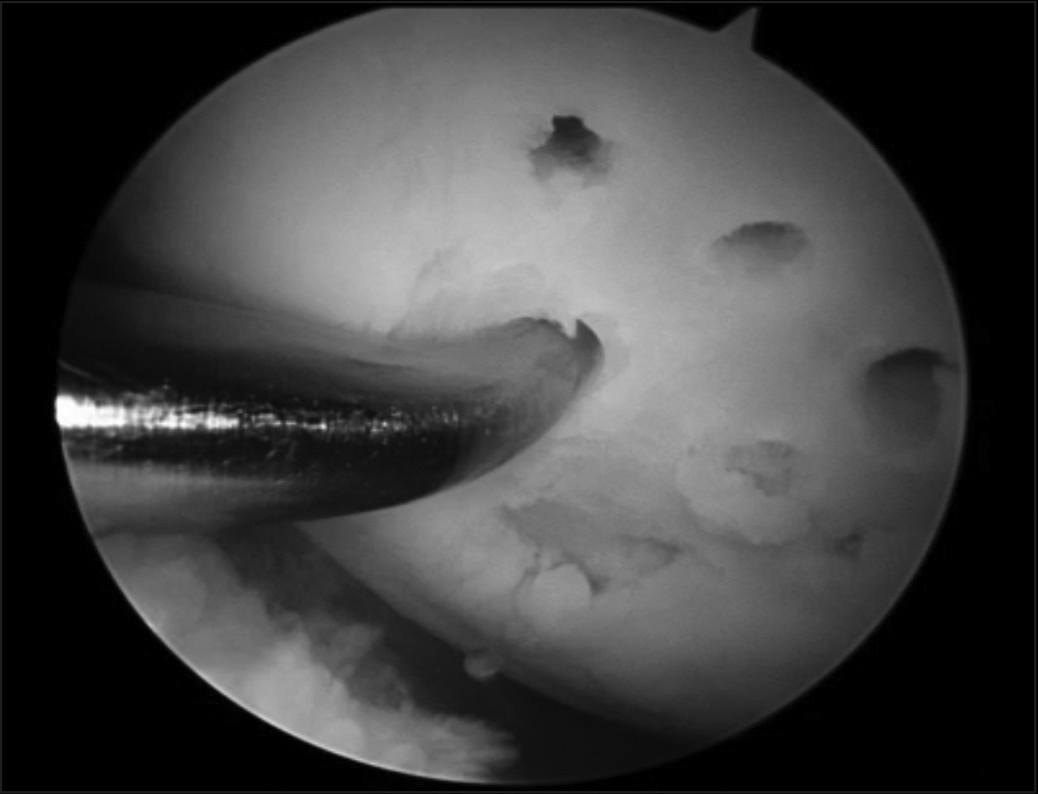
Arthroscopic view of the microfracture process

These techniques also rely on the stimulation of the underlying bone marrow, resulting in fibrocartilage growth. After curetting the OCL down to subchondral bone, a tapered awl can be used to produce the microfractures, approximately 3mm in depth and 3–5mm apart (Fig 3).^25^ The microfractures result in a blood clot containing mesenchymal cells that then form a fibrocartilaginous repair.

In a review article published in 2009, Mithoefer *et al* provided a systematic analysis of 28 studies of over 3,000 patients with an average follow-up duration of 41 months.^26^ It showed that microfracture provided effective short-term functional improvement of knee function but there were poor long-term results. The other shortcomings revealed included poor hyaline repair, variable cartilage volume and long-term functional deterioration.

### Osteochondral autograft/allograft transfer (mosaicplasty)

Osteochondral autograft transfer (OAT) works by removing several plugs of hyaline cartilage and the underlying subchondral bone from an unaffected, non-weight bearing area of the knee. These are used as autograft implants and plugged into the chondral defect. There are several problems with the OAT procedure, the main one being that the topography of the donor site does not match the recipient site and will therefore change the biomechanics and loading. In 2010 Solheim *et al* published a long-term follow-up study on 69 patients with OAT showing good results up to 9 years after surgery.^27^ However, in a randomised controlled trial, Bentley *et al *showed that mosaicplasty was markedly inferior to ACI.^12^

Although very similar to OAT, osteochondral allograft transfer (OALT) does not rely on a donor site but on a cadaveric donor. OALT will theoretically have a like-for-like replacement with no donor site morbidity. It should be biomechanically and topographically similar. Results have shown good to excellent outcomes in up to 80% of cases in some reports and larger defects can be filled.^28^ Apart from having to be an open procedure, OALT carries the disadvantages of rejection, viral disease transmission and tissue availability. In 2009 Birman *et al* investigated the use of humeral heads for OALT on femoral condyles as they can be similar topographically.^29^ The drawback found in their research is that only small grafts can be harvested due to mismatching.

### First generation ACI

In 1994 Brittberg *et al* described the use of ACI in treating full-thickness AC defects of the human knee.^30^ This was achieved with a two-stage procedure. Stage 1 involved arthroscopic biopsy of healthy AC and culture of the chondrocytes to produce between 5 and 10 million cells over a period of 4–6 weeks. Stage 2 involved debridement of the OCL and coverage by a periosteal flap followed by open implantation of these cells into the defect.

**Figure 4:**
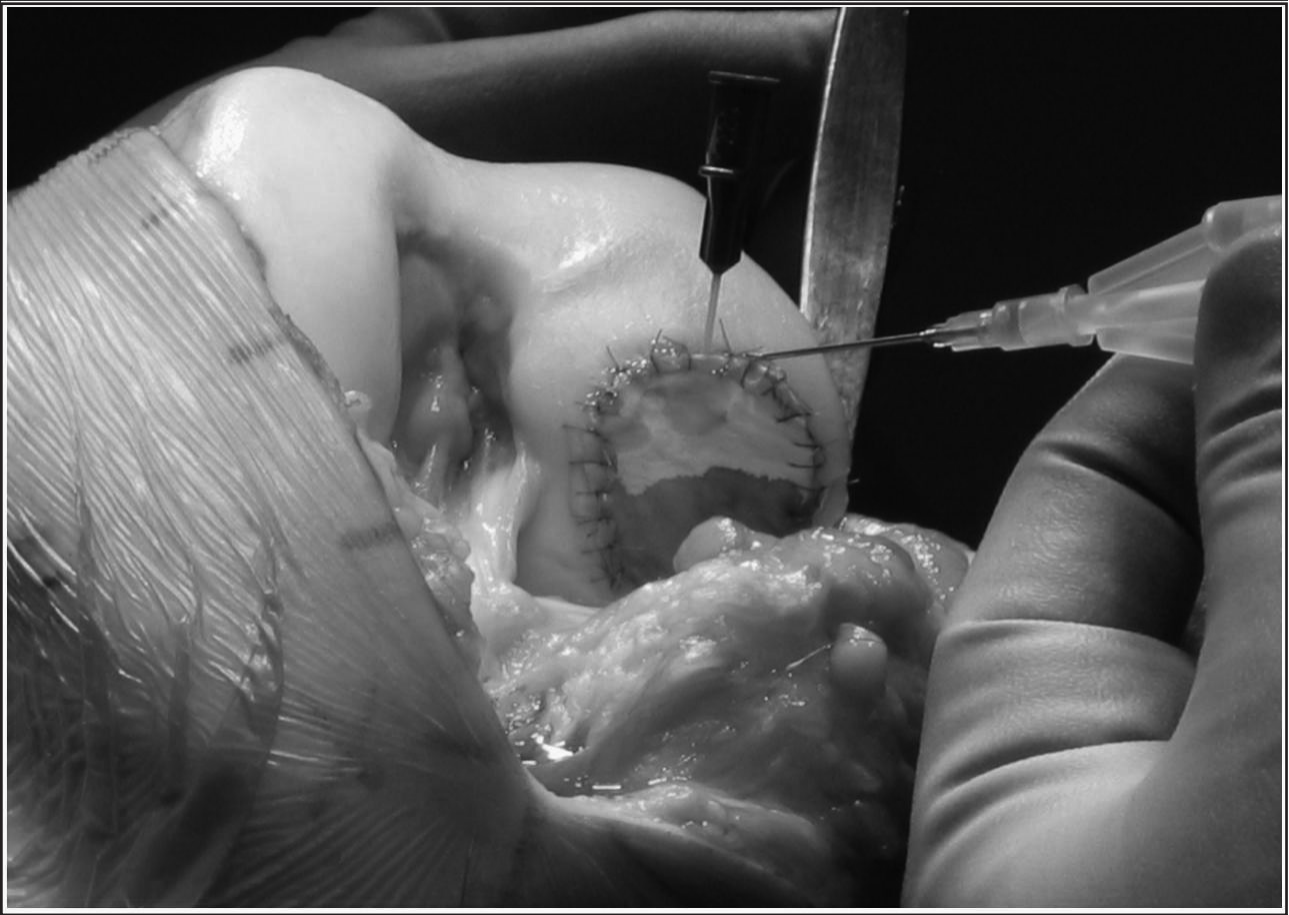
Autologous chondrocyte implantation in a medial femoral condyle, demonstrating the injection of chondrocytes in suspension under a collagen type I/III membrane. The extent of the filling can be seen by the ‘tidemark’ on the membrane, produced by the liquid suspension.

The periosteum is sutured with fine sutures and sealed with fibrin glue to make a watertight seal. The cultured cells are injected beneath it into the OCL (Fig 4). ACI has shown encouraging results. In 2002 Peterson *et al* examined the durability of ACI grafts, showing 84% had ‘good’ to ‘excellent’ results at 5–11 years.^31^ One year later, they evaluated treatment of osteochondritis dissecans with ACI, revealing a 90% successful clinical result.^32^

Use of periosteum proved problematical as hypertrophy of the membrane producing painful clicking occurred in 25% of patients, who required arthroscopic resection. In a randomised controlled trial, Gooding *et al* demonstrated the superiority of a type I/III porcine collagen membrane matrix as a cover for the graft.^33^

In comparative studies, ACI has been evaluated against debridement, microfracture and mosaicplasty. Visna *et al* directly compared ACI and abrasive techniques, showing that even though early intervention is important, ACI significantly improved Lysholm and International Knee Documentation Committee scores over abrasion techniques.^34^ Browne *et al* demonstrated that those patients treated with ACI had a statistically significant improvement of symptoms compared with the microfracture group.^35^ By contrast, Knutsen *et al* showed no difference between microfracture and ACI at five years.^36^ Dozin *et al* compared mosaicplasty with ACI in a randomised controlled trial showing no significant difference.^37^

In a randomised controlled trial, Bentley *et al* compared ACI, using a collagen I/III membrane, with mosaicplasty and showed good or excellent results in 88% versus 69% in the mosaicplasty group.^12^ They also demonstrated superior International Cartilage Repair Society scores with 84% of patients in the ACI group having grade I or II compared with only 35% of the mosaicplasty group.

### Matrix assisted chondrocyte implantation

**Figure 5:**
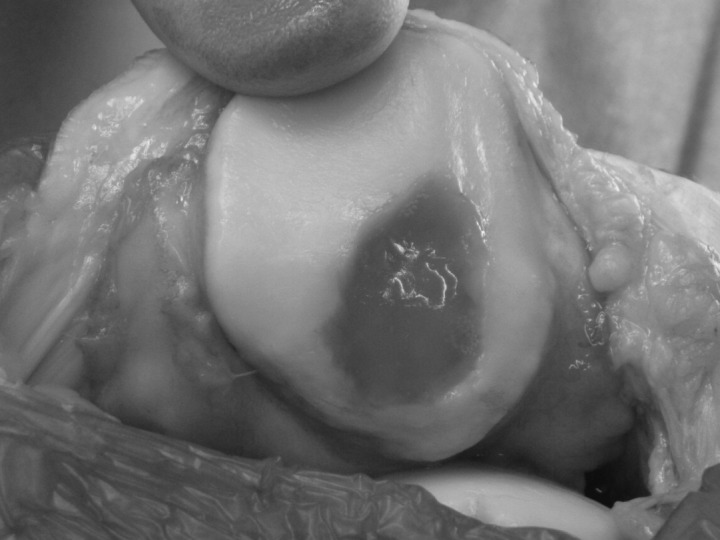
Matrix assisted chondrocyte implantation in a lateral patella facet. The scaffold is held in place with fibrin glue.

Matrix assisted chondrocyte implantation (MACI) uses a scaffold, which is a type I/III collagen. It is also described as second generation ACI. In this process, the scaffold is used to provide a matrix preimplantation (Fig 5). This eliminates the need for a periosteal patch and all the morbidity associated with patching.^33,38,39^ The cells are cultured on the surface of the scaffold, which is then implanted into the defect and secured with fibrin glue. This speeds up the procedure greatly but has the potential disadvantage of a much lower number of implanted cells, up to five times fewer.^13,15^

Saris *et al* used phenotypically selected chondrocytes optimised for their production of proteoglycans and presumed repair capacity.^40^ They showed that in the short term the clinical outcome between microfracture and ACI was similar but ACI showed superior tissue regeneration. In a randomised trial, this method showed a significantly better clinical outcome compared with microfracture^41^ but no comparison with standard ACI or MACI has been reported. Moreover, this technique costs approximately three times more than the standard technique.

Several papers have studied the differences between microfracture and MACI. In 2009 Kon *et al* compared second generation ACI, using a hyaluronan scaffold, and microfracture.^42^ They had 40 patients from each treatment with a minimum follow-up period of five years. Both groups showed a satisfactory outcome at five years but the MACI group showed improved outcome clinically and in terms of return to sporting activity.

### Three-dimensional scaffolds

A three-dimensional scaffold mimicking cartilage structure (‘third generation ACI’) is now being used to provide an increased surface area-to-volume ratio for cellular migration, adhesion and differentiation. As seen in first generation ACI series, the results can be variable. Capito and Spector tried to improve on this by introducing a three-dimensional environment into which to seed the cultured chondrocytes.^43^ They can be made from fibres, sponges or gels and, after the integration of chondrocytes, can be used as a scaffold for implantation.^44^

The scaffolds can be made from either natural or synthetic materials and they generally have similar properties. They should have a degree of porosity to allow integration with the surrounding AC, which will also assist cell migration as well as nutrient and waste product passage, with the optimum pore size being 100–500µm.^45,46^ These products should also degrade non-toxically and keep their stability until the new AC is formed.^47^

## Mesenchymal stem cells

MSCs are multipotent stem cells that can differentiate into a variety of cell types. They have been shown to differentiate into bone, cartilage, fat, marrow, muscle, skin and tendon. Any of these tissues are therefore potential sources of MSCs.^48,49^ Vidal *et al* showed that the chondrogenic potential of bone marrow is greater than that of adipose tissue.^50^ More recently, Fan *et al* described using synovial-derived MSCs for chondrogenesis.^51^ They demonstrated that these cells can be used in cartilage tissue engineering and they use growth factors, outlined below, to increase their potential.

Unfortunately, there is a lack of studies investigating the use of MSCs in the treatment of OCLs. Animal models have been employed and, more recently, Zscharnack *et al* have used an ovine model.^52^ They compared predifferentiated MSCs, undifferentiated MSCs, cell-free and controls using a MACI-like system of hydrogel scaffolds and MSCs instead of chondrocytes. After six months the predifferentiated MSCs showed significantly better histological scores with features of hyaline cartilage.

In an observational cohort study from 2010, Nejadnik *et al* showed that bone marrow-derived MSCs are as effective as chondrocytes for AC repair.^53^ The authors also stated that this method reduces the number of knee operations to one and removes donor site morbidity.

## Growth factors

Growth factors have been a more recent addition to the management of OCLs and cartilage engineering. MSCs and chondrocytes are influenced by a number of different proteins including growth factors. MSCs have the ability to differentiate into a number of different tissue types. Growth factors, among other things, have the ability to influence this differentiation and are currently being investigated. Similarly to stem cells, growth factors are in the very early stages of investigation.

## Conclusions

ACI has become a popular technique for treating isolated chondral defects of the knee and has now been performed on an estimated 35,000 patients worldwide.^54^ Most investigators have reported ‘good’ to ‘excellent’ clinical and histological results using this technique^12,30,31,36,40,54–58^ but there is still scepticism among some surgeons about its effectiveness, the type of repair produced and its durability. Which patients should receive ACI and the timing of its use in relation to other techniques such as microfracture remain somewhat unclear.

From a review of the available literature and our experience of the clinical outcome for isolated chondral defects of the knee in over 1,000 cases using ACI, we draw the following conclusions:

> There is no current evidence to justify treatment in asymptomatic, very small (<1cm^2^) chondral defects of the knee.
> Adult patients with symptomatic full-thickness defects have poor results if not treated.
> Instability and malalignment require correction.
> Smaller (<1cm^2^), well contained lesions may be suitable for microfracture but for patients who have larger defects, ACI is a satisfactory procedure in 70–80% of cases.
> Motivated patients aged 15–55 years with a single lesion and a short (<1 year) history and no previous procedures have the best outcome.
> ACI leads to a statistically significant improvement in objective and patient reported clinical outcome scores and produces a durable outcome for as long as ten years. The clinical results of the ACI and MACI techniques are comparable and the percentage of hyaline cartilage at biopsy appears to improve with time.
> Lesions of the femoral condyles have superior results to those in the patellofemoral joint.^59^

## Future trends

OCLs present a therapeutic challenge for a number of different reasons. The main difficulties are due to the poor healing potential of AC, its response to injury and constant mechanical loading, and potential disruption of any reconstruction by joint movement and load bearing stresses.

Research into OCLs over 40 years has progressed into multiple fields. The main issue with converting the research into clinical practice is the lack of long-term, evidence-based studies although there are a number of reasons for this. The rapid development of this field has proven sometimes to be detrimental to its own progression. There is such a variety of options giving similar reported outcomes with pain-free repair of OCLs over short periods that selection of patients can be problematic. The preliminary nature of this research also means that it is being spread over a large field. When several types of repair have been shown to be more statistically significant than others, the research can then be focused, leading to long-term validated studies.

Currently, primary arthroscopy is used to identify and document an OCL and to harvest chondrocytes for culture. If MSCs (from bone marrow) can be used to create chondrocytes and MRI continues to evolve into a more sensitive and specific investigation, this primary arthroscopic procedure can be avoided and replaced by a single arthroscopic procedure, reducing the overall morbidity of the reconstruction. MRI is also becoming more common in monitoring progress and repair of procedures so that this could negate the need for a follow-up arthroscopic biopsy.

Reviewing the latest tissue engineering studies has shown that there may not be one specific scaffold that has superior properties compared with the others. Many researchers are now concentrating on combining several different types of scaffolds and growth factors. This is designed to mimic the structural and environmental components of cartilage more accurately, theoretically providing a superior repair.

The future of autologous chondrocyte transplantation relies heavily on biomedical research. Once all the different elements of the scaffold can be optimised, they can be applied clinically. The ideal scaffold should have a three-layer, three-dimensional structure similar to AC, incorporating growth factors and chondrocytes. This should then be applied easily to an OCL and held in position in one simple procedure. Nevertheless, at this time, ACI and its modifications give the best chance of relieving painful osteochondral injuries and preventing ‘early onset’ osteoarthritis.
